# Flexible Loading Phase Treat-and-Extend Regimen with Faricimab for Neovascular Age-Related Macular Degeneration: A Real-World Study †

**DOI:** 10.3390/biomedicines13122909

**Published:** 2025-11-27

**Authors:** Akira Machida, Junko Kurihara, Yuki Hirata, Eriko Machida, Ryuya Murakami, Akari Oka, Ai Yoneda, Eiko Tsuiki, Akio Oishi

**Affiliations:** 1 Department of Ophthalmology and Visual Sciences, Graduate School of Biomedical Sciences, Nagasaki University, Sakamoto 1-7-1, Nagasaki 852-8102, Japanakio.oishi@nagasaki-u.ac.jp (A.O.); 2 Department of Ophthalmology, The Japanese Red Cross Nagasaki Genbaku Hospital, Mori-machi 3-15, Nagasaki 852-8511, Japan

**Keywords:** faricimab, flexible loading phase, neovascular age-related macular degeneration, polypoidal choroidal vasculopathy, real-world study, treat-and-extend

## Abstract

**Background/Objectives**: We aimed to evaluate the efficacy of a flexible loading-phase treat-and-extend regimen using faricimab, in which the number of loading-phase intravitreal injections was tailored to individual disease activity. **Methods**: This observational cohort study included 50 treatment-naïve eyes with neovascular age-related macular degeneration, treated with faricimab in Japan; approximately half of the eyes had polypoidal choroidal vasculopathy (PCV). Disease activity after one injection was assessed at the second visit (4 weeks later) to determine the treatment interval for subsequent injections. The primary outcome measure was the injection interval and visual/anatomical outcomes at 1 year after treatment initiation. **Results**: Of the 50 eyes, 43 completed a 1-year follow-up, including 27 eyes with PCV. The mean logarithm of the minimum angle of resolution best-corrected visual acuity improved from 0.35 ± 0.32 to 0.19 ± 0.3 over 1 year. Overall, 60.5% achieved 16-week intervals, and 74.4% reached intervals of ≥12 weeks. A shorter loading phase (two or three injections) was associated with fewer total injections and higher rates of fluid resolution, without compromising visual outcomes. The presence of PCV and ellipsoid zone disruption were identified as risk factors for failure to extend treatment intervals beyond 16 weeks. **Conclusions**: A flexible loading-phase treat-and-extend regimen using faricimab yields outcomes comparable to those of the TENAYA protocol, with fewer injections, despite the high proportion of eyes with PCV. This simple approach is straightforward in design and may reduce treatment burden while maintaining efficacy.

## 1. Introduction

Age-related macular degeneration (AMD) is one of the leading causes of blindness in developed countries [1,2]. Anti-vascular endothelial growth factor (VEGF) therapy has markedly improved the visual outcomes of macular neovascularization (MNV) secondary to AMD [3,4,5,6]. However, anti-VEGF agents are not curative and require maintenance, thus incurring substantial financial and logistical burdens [7]. Agents that offer greater durability (e.g., brolucizumab, faricimab, and high-dose aflibercept) than conventional options may help alleviate this treatment burden. In comparison with older therapies, these newer agents enable extended dosing intervals and reduced injection frequencies [8,9,10].

Faricimab is the first bispecific IgG1 monoclonal antibody that targets both VEGF-A and angiopoietin-2 (Ang-2) [11]. Its extended durability has been demonstrated in pivotal trials using a fixed dosing regimen, with intravitreal administration up to every 16 weeks [9]. However, the necessity of this fixed loading strategy for faricimab—which has a distinct pharmacological profile and longer duration of action—remains uncertain. Many patients also maintain favorable outcomes with extended intervals [12,13], suggesting that the necessity of a fixed loading phase should be further investigated.

Thus, the objective of this study was to assess the efficacy of a flexible loading-phase treat-and-extend (TAE) protocol using faricimab in a japanese cohort of patients with neovascular AMD (nAMD). Additionally, we performed exploratory analyses, while acknowledging the limited sample size, to identify subgroups most likely to benefit from faricimab and explored baseline clinical factors that may support drug selection and the development of personalized treatment strategies for patients with nAMD.

## 2. Materials and Methods

### 2.1. Study Design and Participants

This was a non-randomized, open-label, prospective observational study conducted under real-world clinical conditions. A total of 50 consecutive eyes from 50 patients met the eligibility criteria and were enrolled at Nagasaki University Hospital and Nagasaki Genbaku Hospital between September 2022 and December 2023. Eyes that discontinued the study, withdrew consent, or deviated from the protocol were excluded from the final analysis.

The inclusion criteria were: (1) age ≥ 50 years; (2) presence of MNV; and (3) initiation of faricimab treatment during the study period. Exclusion criteria were: (1) axial length ≥ 26.5 mm; (2) presence of inflammatory or genetic conditions associated with MNV; (3) history of prior treatment for nAMD; (4) presence of other retinal or optic nerve disorders; and (5) prior intraocular surgery other than cataract extraction. For each participant, only one eye was selected for analysis. In participants in whom both eyes received treatment, the first treated eye during the study period was selected. Clinicodemographic characteristics (age, sex, and medical history) were obtained from baseline interviews and medical records. Comorbidities included hypertension, diabetes mellitus, malignancy, cardiovascular events, anticoagulant use, and smoking history.

### 2.2. Treatment Protocol

All participants received intravitreal faricimab as part of routine clinical care under a flexible TAE strategy, in which the number of injections during the loading phase was adjusted according to disease activity. Each participant received an initial intravitreal injection of faricimab, and disease activity was assessed at the second visit (4 weeks later) using SD-OCT and fundus examination. Subsequent injection intervals were adjusted according to the following criteria, applied at the discretion of the treating ophthalmologist: (1) extension: complete resolution of both SRF and intraretinal fluid (IRF), with no new retinal hemorrhage; (2) maintenance: presence of residual SRF or IRF without a ≥50 μm increase in CRT at the fovea with reference to prior OCT, and no new retinal hemorrhage; and (3) shortening: increase in SRF or IRF, with a CRT increase >50 μm or evidence of new retinal hemorrhage.

Injection intervals were adjusted in 4-week increments. To accommodate scheduling variability inherent in the real world, visits occurring within ±2 weeks of the planned interval were considered protocol-compliant and rounded to the nearest multiple of 4 weeks for analysis. Intervals deviating by more than ±2 weeks from the planned schedule were defined as protocol deviations and excluded from analysis. Under the Japanese national health insurance system, injection intervals <8 weeks are not reimbursable after the initial loading series of faricimab. Therefore, when administering the fifth injection at an interval of ≥8 weeks was not possible, treatment was switched to intravitreal aflibercept (2 mg), with subsequent injections adjusted according to the same TAE protocol. In participants with suboptimal response to aflibercept, switching to brolucizumab was permitted at the treating ophthalmologist’s discretion.

### 2.3. Assessments

Ophthalmologic evaluations were performed at baseline and during each follow-up visit. Assessments included BCVA measured using a Landolt C chart, with values converted to logarithm of the minimum angle of resolution (logMAR) units for statistical analysis; axial length measurement (IOLMaster 500; Carl Zeiss Meditec, Dublin, CA, USA); color fundus photography (AFC-230; Nidek, Gamagori, Japan); SD-OCT; fluorescein and indocyanine green angiography (Spectralis; Heidelberg Engineering, Heidelberg, Germany); and OCT angiography (OCT-S1; Canon, Tokyo, Japan; and Avanti; Optovue, Fremont, CA, USA).

The nAMD subtype was diagnosed and classified based on findings on multimodal imaging, including SD-OCT, fluorescein and indocyanine green angiography, and OCT angiography. Fluorescein and indocyanine green angiographies were obtained before or immediately after treatment initiation, unless contraindicated. The nomenclature for nAMD subtypes followed previously published criteria [14]. In this study, type 1 MNV with polyps was defined as PCV irrespective of pachychoroid characteristics [15]. SD-OCT imaging included 30° horizontal scans through the fovea (both standard and enhanced depth imaging) and 15 raster scans covering a 20° × 15° rectangular region. Subretinal hemorrhage (SRH) was defined as hemorrhage within 6 mm radius from the foveal center, confirmed by SD-OCT and fundus photography. Hyperreflective foci (HRF) and subretinal hyperreflective material (SHRM) were evaluated within the same radius. HRF were defined as discrete hyperreflective spots in the neurosensory retina, while SHRM was hyperreflective material beneath the retina.

Central choroidal thickness (CCT) was determined at the foveal center as the vertical distance from the outer border of Bruch’s membrane to the inner scleral boundary. Pigment epithelial detachment (PED) height was defined as the vertical distance between the outer surface of the retinal pigment epithelium and inner surface of Bruch’s membrane at the foveal center. Ellipsoid zone (EZ) integrity was assessed on both vertical and raster scans within a 1 mm radius from the foveal center. All measurements were independently performed by two graders (Machida E. and Kurihara J.) masked to clinical outcomes.

### 2.4. Main Outcome Measures

The primary outcome measure was achieving an injection interval of ≥16 weeks at the final follow-up visit, occurring approximately 1 year after treatment initiation, specifically between weeks 40 and 48. The visit occurring between weeks 20 and 28 was designated as the 6-month time point for interim evaluation. For participants who were switched to an alternative anti-VEGF agent, injection intervals were calculated from the date of the initial faricimab injection using the same method.

### 2.5. Statistical Analysis

Continuous variables are expressed as means ± standard deviations (SD). Group comparisons were conducted using *t*-tests or one-way ANOVA, and categorical variables were compared with Fisher’s exact test. Logistic regression analysis was conducted to identify predictors of successful treatment interval extension beyond 16 weeks, with extension success as the dependent variable. Multiple linear regression was performed to identify factors associated with the number of injections administered during the loading phase. Independent variables included age; sex; presence of polypoidal lesions; baseline BCVA; systemic conditions (hypertension, diabetes mellitus, cardiovascular disease, anticoagulant use, history of malignancy, and smoking); CRT; CCT; PED height; presence of SRH, SHRM, HRF; and EZ integrity. Data analyses were conducted using Eazy R [16], a graphical interface for the biostatistics-specific R Commander (version 4.2.0, R Foundation for Statistical Computing, Vienna, Austria). A *p* value < 0.05 was considered significant. Because this study aimed to explore the feasibility of a flexible loading-phase TAE regimen in this limited Japanese cohort, rather than to test a predefined hypothesis, no formal power or sample size calculation was performed. The sample size was determined by the number of consecutive eligible patients during the study period.

## 3. Results

**Figure 1:**
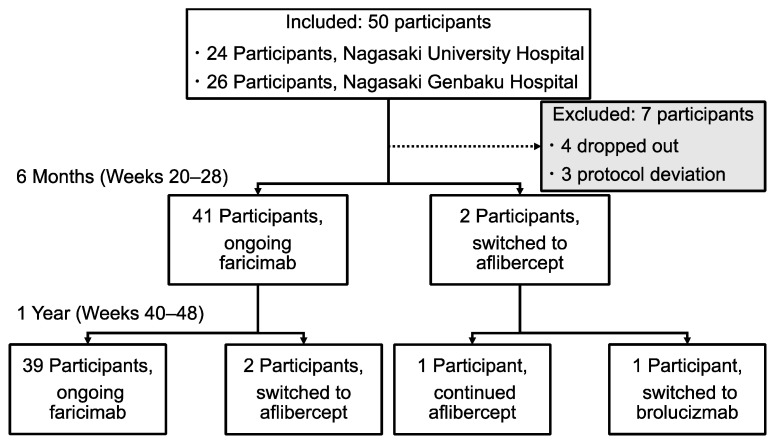
Flowchart of participant allocation and treatment course over 1 year. Overall, 50 participants with neovascular retinal disease were enrolled across two institutions (24 from Nagasaki University Hospital and 26 from Nagasaki Genbaku Hospital). Seven participants were excluded due to dropout (*n* = 4) or protocol deviation (*n* = 3), leaving 43 participants for analysis. At 6 months (weeks 20–28), 41 participants continue faricimab treatment, whereas 2 were switched to aflibercept (2 mg). At 1 year (weeks 40–48), 39 participants remained on faricimab, and 2 participants were transitioned to aflibercept. Among them, 1 participant continued on aflibercept, whereas the other was subsequently switched to brolucizumab.

Overall, 50 eyes were included. Figure 1 illustrates the study population and treatment course throughout the observation period. During the first 6 months, treatment was discontinued in four eyes due to participant refusal or loss to follow-up, and the predefined protocol was not followed in three additional eyes owing to delays in scheduled visits. These 7 eyes were excluded from the final analysis. Among the remaining 43 eyes, treatment in two eyes was switched to aflibercept prior to the 6-month visit due to insufficient anatomical response. Between months 6 and 12, an additional two eyes were transitioned to aflibercept, and one of these was subsequently switched to brolucizumab due to persistent exudative activity. Ultimately, 43 eyes completed the 12-month observation period and were included in the final analysis. Of these, 39 eyes remained on faricimab throughout the entire study duration.

The baseline characteristics of the 43 included eyes, along with their distribution by disease subtype, are summarized in Table 1. The mean (±SD) age was 76.6 ± 8.9 years, and 12 participants (27.9%) were female. The mean baseline best-corrected visual acuity (BCVA) was 0.35 ± 0.32 logMAR. With respect to disease subtypes, 13 eyes were classified as typical type 1 MNV; 26 eyes, polypoidal choroidal vasculopathy (PCV); 3 eyes, typical type 2 MNV; and 1 eye, retinal angiomatous proliferation. IRF involving the fovea, subretinal fluid (SRF), and pigment epithelial detachment (PED) were present at baseline in 32.6%, 83.7%, and 72.1% of the eyes, respectively.

**Figure 2:**
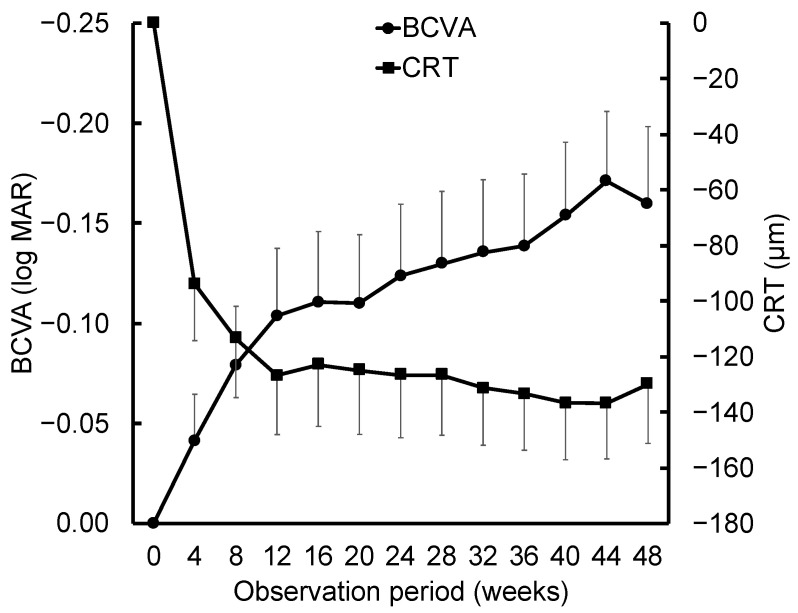
Changes in best-corrected visual acuity (BCVA) and central retinal thickness (CRT) over 48 weeks. The mean BCVA (logMAR, indicated by circles) and CRT (μm, indicated by squares) are assessed at baseline and up to 48 weeks. BCVA demonstrated gradual improvement, while CRT exhibited a sharp reduction by week 4, which was maintained throughout the observation period. To maintain clarity, the error bars depict the standard error of the mean instead of the standard deviation. Data are analyzed using the last observation carried forward (LOCF) method for participants with missing follow-up data.

Figure 2 illustrates the clinical course of BCVA and central retinal thickness (CRT), based on the last observation carried forward method, over the 1-year observation period. BCVA improved by 0.13 ± 0.24 logMAR at 6 months and further improved to 0.16 ± 0.26 logMAR at 1 year. CRT decreased by 124.8 ± 143.7 µm at 6 months and by 126.1 ± 139.3 µm at 1 year. The overall mean number of injections during the 1-year follow-up was 6.6 ± 1.5. At the 1-year time point, 32 eyes (74.4%) achieved an injection interval of ≥12 weeks, and 26 eyes (60.5%) reached an interval of ≥16 weeks. The 6-month and 1-year dry rates (i.e., complete resolution of exudative findings on optical coherence (OCT)) were 83.7% (36 eyes) and 81.4% (35 eyes), respectively.

A total of 17 participants (39.5%) were eligible for interval extension at the second visit, 10 participants (23.3%) at the third visit, and 16 participants (37.2%) required four loading injections in accordance with the conventional protocol. The baseline characteristics and clinical outcomes of these subgroups are summarized in Table 2. Over the 1-year follow-up, eyes that completed the loading phase with fewer injections required significantly fewer total injections overall while achieving similar or better anatomical and functional outcomes. Both the 2- and 3-injection groups demonstrated dry macula rates and proportions of eyes reaching extended treatment intervals (≥12 or ≥16 weeks) that were comparable to or exceeded those observed in the 4-injection group. Significant differences in baseline characteristics were identified across groups: the 2-injection group tended to have lower baseline CRT and a lower frequency of PCV and SRF. Multiple regression analysis demonstrated that the presence of PCV significantly associated with an increased number of injections during the loading phase, with an average increase of 0.7 injections (*p* < 0.001).

**Figure 3:**
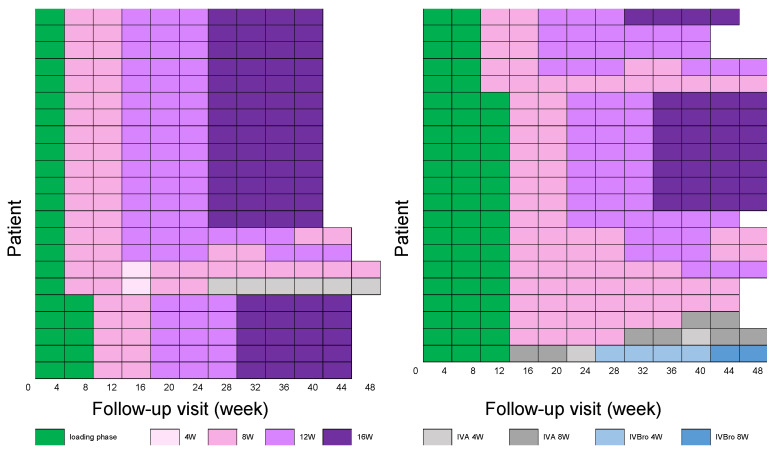
Injection intervals and treatment transitions over the 48-week observation period. Each horizontal row represents a single participant, with color-coded bars denoting injection intervals and treatment types. Green indicates the faricimab loading phase, followed by scheduled intervals of 4, 8, 12, or 16 weeks (shades of pink to purple). Gray and blue segments depict transitions to aflibercept 2 mg (IVA) and brolucizumab (IVBro), respectively, with corresponding injection intervals. This figure illustrates individualized treatment modifications during the maintenance phase.

**Figure 4:**
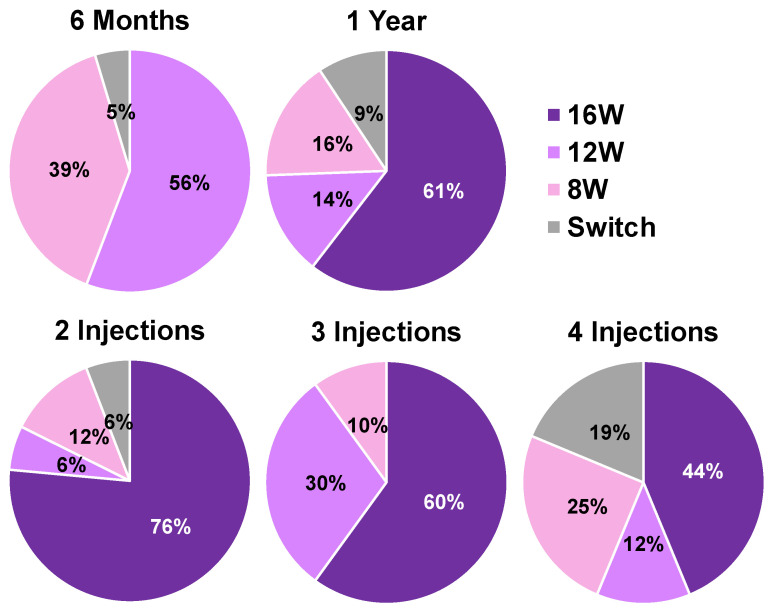
Distribution of injection intervals at 6 months and 1 year. The upper panels show pie charts displaying the proportion of participants by injection interval among those who remained on faricimab therapy. At 6 months, 56% achieved a 12-week interval, 39% remained on an 8-week interval, and 5% were transitioned to another agent. At 1 year, 61% reached intervals beyond 16 weeks, 14% remained on a 12-week interval, 16% on an 8-week interval, and 9% were switched to other agents. These findings demonstrate a progressive extension of treatment intervals over time in most participants. The lower panels show the distribution of treatment intervals at 1 year according to the number of loading injections (2, 3, or 4). The proportion of participants achieving intervals beyond 16 weeks decreasing as the number of loading injections increased.

Figure 3 illustrates injection intervals and treatment transitions over the 48-week observation period for each participant. During the treatment course, 10 participants (23.3%) required shortening of their injection interval due to insufficient response. Figure 4 displays the distribution of injection intervals at 6 months and 1 year among participants who continued treatment with faricimab. At 6 months, 55.8% were on a 12-week interval, 39.5% on an 8-week interval, and 4.7% had switched to another anti-VEGF agent. At 1 year, 60.5% had achieved injection intervals ≥16 weeks, whereas 14.0% and 16.3% remained on 12- and 8-week intervals, respectively. Additionally, 9.3% of the participants required a switch to an alternative anti-VEGF agent due to insufficient response or recurrent disease activity. When analyzed according to the number of loading injections, the proportion of participants achieving injection intervals ≥16 weeks at 1 year decreased as the number of loading injections increased.

Participants who achieved an injection interval of ≥16 weeks at the 1-year follow-up were defined as the successful group. Their baseline characteristics are summarized in Table 3. Logistic regression analysis identified the presence of polypoidal lesions (odds ratio: 0.101, 95% confidence interval: 0.019–0.545, *p* = 0.008) and baseline EZ disruption (odds ratio: 0.075, 95% confidence interval: 0.012–0.477, *p* = 0.006) as significant risk factors for failure to achieve the 16-week interval. No systemic adverse events were reported in any of the 50 eyes throughout the treatment period. Recurrent sub-retinal hemorrhage was observed in only one patient.

## 4. Discussion

The current standard anti-VEGF treatment strategy for nAMD typically begins with a loading phase comprising three initial intravitreal injections. This approach was first formalized in the PIER and EXCITE studies [17,18], which implemented fixed, monthly dosing over 3 months followed by interval extension. Although the term “loading phase” was not explicitly defined in these trials, they established the clinical rationale for initiating therapy with intensive dosing. The PrONTO study further expanded this treatment paradigm by introducing OCT-guided retreatment with ranibizumab, laying the foundation for individualized anti-VEGF regimens [19]. Subsequent studies demonstrated that pro re nata (PRN) protocols incorporating an initial loading phase result in superior visual outcomes compared to PRN regimens without induction [20]. The TAE regimen has since become widely adopted, allowing adjustment of treatment intervals based on disease activity after the loading phase [21]. Multiple large-scale clinical trials have validated this strategy, reporting reduced treatment burden and improved visual acuity outcomes compared to that of PRN approaches [22,23]. In pivotal trials evaluating faricimab, a fixed four-injection loading phase was followed by personalized dosing intervals ranging from 8 to 16 weeks, guided by anatomical and functional disease activity [9]. These studies demonstrated non-inferiority in visual outcomes compared to bimonthly aflibercept. However, considering faricimab’s extended duration of action and dual-target mechanism, the clinical need for a conventional fixed loading phase originally established for shorter-acting agents warrants reconsideration. Ongoing challenges related to overtreatment and undertreatment, which are driven by heterogeneous disease expression and therapeutic response, underscore the need for regimen optimization tailored to individual characteristics.

Our study evaluated a flexible loading-phase TAE regimen using faricimab, wherein the number of loading-phase intravitreal injections was adjusted based on each individual’s early treatment response. The findings suggest that this personalized approach may reduce treatment burden without compromising visual or anatomical outcomes, highlighting the potential for more tailored treatment protocols in real-world clinical practice.

A notable characteristic of the study population was that approximately half of the participants had type 1 MNV with polyps, a common nAMD subtype among Japanese patients [24]. In our cohort, 60% of the included eyes were classified as PCV, which may appear disproportionate compared with Western populations. However, this distribution closely reflects the epidemiological characteristics of Japanese patients with nAMD, in whom PCV accounts for approximately 50–60% of treatment-naïve cases, and tends to be more prevalent in younger patients in real-world practice. Therefore, the current sample composition is consistent with real-world demographic patterns in East Asian populations. Ongoing investigations and preclinical studies continue to explore the role of Ang-2 inhibition in promoting vascular stabilization [25,26], and faricimab has demonstrated promise in stabilizing polypoidal lesions, as evidenced by a high rate of polyp regression reported in previous studies [27]. However, in the present study, eyes with PCV required more injections during both the loading phase and 12-month period than did those with non-PCV subtypes. All four eyes that required switching to another anti-VEGF agent were in the PCV group. PCV was also identified as a significant risk factor for failure to achieve an injection interval of ≥16 weeks at the 1-year evaluation. In all cases requiring a switch to another anti-VEGF agent, none of the participants could achieve an injection interval longer than 8 weeks during the first year. With the recent approval of aflibercept 8 mg, the therapeutic options for refractory cases have further expanded, underscoring the need to reconsidering appropriate rescue regimens.

We previously reported that switching from aflibercept to faricimab resulted in improved treatment durability in non-PCV subtypes [28], and the efficacy of faricimab for non-PCV subtypes was similarly demonstrated in the present study. However, considering that clinical responsiveness does not always parallel pathological processes, we cannot conclude that faricimab should be avoided in PCV. In a prior study involving 47 individuals with baseline SRH, 24 experienced recurrent hemorrhage over 2 years, with 12 of them having PCV [29]. In the current cohort, 18 eyes presented with SRH at baseline, but only 1 eye with PCV developed hemorrhage during the treatment period. This suggests that faricimab may have helped stabilize polypoidal lesions. Large-scale studies have similarly reported a lower incidence of SRH during nAMD treatment with faricimab than with other anti-VEGF agents [30]. Further long-term investigations are warranted to determine the optimal anti-VEGF agent by disease subtype.

In the TENAYA trial (*n* = 334) [9] and its Japanese subgroup analysis (*n* = 26) [31], the mean visual acuity improvements were 5.8 and 6.5 letters, respectively, corresponding to approximately 0.12 and 0.13 logMAR. In our cohort, the mean improvement reached 0.16 logMAR, comparable to or slightly greater than those reported in the trial. Although the peak improvement in visual acuity in our study occurred later than in clinical trials, the group with fewer loading-phase injections demonstrated a higher dry macula rate and favorable treatment responsiveness. These findings support the view that additional loading injections may have limited necessity in real-world settings. The number of loading-phase injections may not be the primary determinant of long-term outcomes, and early anatomical response more accurately predicts visual prognosis [32].

Regarding anatomical changes, the mean reduction in retinal thickness in the Japanese subgroup of the TENAYA trial [31] was 140.6 μm, whereas it was 126.1 μm in our study. However, the baseline thickness in the TENAYA subgroup was 41.7 µm greater (noting that central subfield thickness rather than CRT was used), suggesting a similar degree of change. For fluid resolution, the 1-year IRF-free rate in TENAYA ranged from 75.5% to 82.1%, whereas it was 95.3% (41/43 eyes) in our cohort. The SRF-free rate in TENAYA was 69.6% to 78.5%, whereas it was 86.0% (37/43 eyes) in our cohort, indicating favorable anatomical outcomes in the real world. At the 1-year time point, 60% of eyes in the cohort achieved a treatment interval <16 weeks, and 75% exceeded 12 weeks. These outcomes aligned with the distribution of faricimab dosing intervals at the 2-year mark in the TENAYA/LUCERNE trials, which adopted a personalized regimen allowing interval modification and extension [33].

There were approximately 6.75 injections over 48 weeks in the Japanese subgroup of the TENAYA trial [31], and the mean number of injections in our cohort during the same period was slightly lower at 6.58. The difference was smaller than expected, likely due to the more flexible protocol in the TENAYA trial compared to the TAE regimen. Furthermore, participants who switched to aflibercept due to inadequate treatment response received between 9 and 11 injections combining faricimab and aflibercept. This may have increased the overall number of injections and limited direct comparability with clinical trial results. Nevertheless, a notable strength of this flexible regimen is its simplicity: treatment intervals are adjusted solely based on disease activity, without reliance on a fixed loading protocol. This strategy supports prompt clinical decision-making, reduces the burden on healthcare staff, and enables efficient management of nAMD even in high-risk PCV eyes. These findings support the flexibility of the TAE regimen in accommodating individual patient response while maintaining comparable outcomes to standard regimens.

### Limitations

This study lacked a control group. The most appropriate comparators would be the Japanese subgroups from the TENAYA trials [31,34]; however, as subsets of large-scale randomized clinical trials, those cohorts differ from ours with respect to inclusion criteria and demographics. Nevertheless, we attempted to mitigate potential biases and enhance the generalizability of our findings by consecutively enrolling all eligible patients who initiated faricimab during the study period and applying a standardized evaluation protocol across the cohort. As a real-world study, our findings are inherently susceptible to selection bias. Although the present regimen was recommended to all treatment-naïve patients with AMD during the study period, some patients with poorer baseline status or who were deemed unsuitable for treatment declined participation. In addition, patients who discontinued follow-up or deviated from the regimen were likely to include those with suboptimal response or lower treatment satisfaction, further contributing to bias. Patient characteristics were primarily self-reported, which may have introduced underreporting or inaccuracies, particularly for variables such as smoking history. Large-scale prospective studies with control groups are warranted to further evaluate the true utility of a flexible loading-phase regimen. In addition, the uneven distribution of AMD subtypes, with a predominance of PCV, may limit the generalizability of the findings. However, this reflects the real-world epidemiology of Asian patients with nAMD, in whom PCV represents more than half of treatment-naïve cases. Further multicenter studies including more balanced subtype representation are warranted to validate these results.

## 5. Conclusions

In conclusion, the loading phase in faricimab for nAMD can be omitted without compromising visual acuity, anatomical outcomes, dry macula rates, or achievable injection intervals. PCV or baseline EZ disruption increases the risk of failing to achieve treatment intervals of ≥16 weeks. This flexible loading-phase TAE regimen using faricimab may effectively reduce treatment burden for both patients and healthcare providers, even in a cohort with a high proportion of PCV eyes, and can thus serve as a practical alternative for managing nAMD in the real world. Moreover, given the dual inhibition of VEGF-A and Ang-2 by faricimab, our findings highlight the importance of integrating molecular mechanisms of vascular stabilization with clinical regimen design. Future translational and mechanistic studies should aim to clarify how differential disease subtypes, such as PCV, modulate responsiveness to Ang-2 blockade. Such investigations may pave the way for biomarker-driven individualized therapy and further optimization of anti-VEGF treatment strategies in nAMD.

## Figures and Tables

**Table 1:** Baseline participant characteristics by neovascular age-related macular degeneration subtype.

	Total	Type 1		Type 2	Type 3
		Without polyps	With polyps		
Number of eyes	*n* = 43	*n* = 13	*n* = 26	*n* = 3	*n* = 1
Clinicodemographic characteristics					
Age (years), mean (SD)	76.6 (8.9)	78.1 (12.4)	75.6 (7.2)	78.1 (8.4)	80.3 (NA)
Sex: Female, *n* (%)	12 (27.9)	3 (23.1)	6 (23.1)	3 (100.0)	0 (0.0)
Hypertension, *n* (%)	29 (67.4)	8 (61.5)	17 (65.4)	3 (100.0)	1 (100.0)
Diabetes mellitus, *n* (%)	8 (18.6)	3 (23.1)	4 (15.4)	1 (33.3)	0 (100.0)
Malignancy, *n* (%)	9 (20.9)	2 (15.4)	6 (23.1)	0 (0.0)	1 (100.0)
History of cardiovascular events, *n* (%)	6 (14.0)	1 (7.7)	3 (11.5)	1 (33.3)	1 (100.0)
Anticoagulant use, *n* (%)	9 (20.9)	3 (23.1)	3 (11.5)	2 (66.7)	1 (100.0)
Smoking history, *n* (%)	23 (53.5)	7 (53.8)	15 (57.7)	0 (0.0)	1 (100.0)
BCVA, logMAR, mean (SD)	0.35 (0.32)	0.32 (0.28)	0.32 (0.33)	0.58 (0.39)	0.70 (NA)
Anatomical parameters at baseline					
CRT (μm), mean (SD)	312.4 (140.5)	251.4 (76.0)	332.4 (164.0)	353.7 (27.4)	460.0 (NA)
CCT (μm), mean (SD)	258.6 (99.1)	240.1 (113.5)	274.3 (93.9)	196.7 (86.6)	277.0 (NA)
PED height at fovea (μm), mean (SD)	67.1 (125.0)	40.2 (39.1)	82.8 (154.9)	10.7 (18.5)	177.0 (NA)
Disrupted EZ at fovea, *n* (%)	28 (65.1)	8 (61.5)	16 (61.5)	3 (100.0)	1 (100.0)
SRH, within 6 mm, *n* (%)	18 (41.9)	4 (30.8)	11 (42.3)	2 (66.7)	1 (100.0)
SHRM within 6 mm, *n* (%)	29 (67.4)	7 (53.8)	19 (73.1)	3 (100.0)	0 (0.0)
HRF within 6 mm, *n* (%)	24 (55.8)	5 (38.5)	17 (65.4)	2 (66.7)	0 (0.0)
Intraretinal fluid in fovea, *n* (%)	14 (32.6)	4 (30.8)	6 (23.1)	3 (100.0)	1 (100.0)
Subretinal fluid in fovea, *n* (%)	36 (83.7)	11 (84.6)	22 (84.6)	2 (66.7)	1 (100.0)
Presence of PED in fovea, *n* (%)	31 (72.1)	10 (76.9)	19 (73.1)	1 (33.3)	1 (100.0)

Values are presented as mean (standard deviation) or number (percentage). CRT: central retinal thickness; CCT: central choroidal thickness; PED: pigment epithelial detachment; EZ: ellipsoid zone; SRH: subretinal hemorrhage; SHRM: subretinal hyperreflective material; HRF: hyperreflective foci; BCVA: best-corrected visual acuity; NA: not available.

**Table 2:** Comparison of baseline characteristics and 1-year outcomes according to the number of loading injections for neovascular age-related macular degeneration.

	Number of Loading Phase Injections			
	2 Injections	3 Injections	4 Injections	*p* values
Number of eyes, *n* (%)	17 (39.5)	10 (23.3)	16 (37.2)	
Clinicodemographic characteristics				
Age (years), mean (SD)	77.6 (8.9)	74.4 (8.8)	77.0 (9.4)	0.656
Sex: Female, *n* (%)	4 (23.5)	4 (40.0)	4 (25.0)	0.685
Hypertension, *n* (%)	12 (70.6)	5 (50.0)	12 (75.0)	0.415
Diabetes mellitus, *n* (%)	4 (23.5)	1 (10.0)	3 (18.8)	0.882
Malignancy, *n* (%)	4 (23.5)	2 (20.0)	3 (18.8)	1.000
History of cardiovascular events, *n* (%)	4 (23.5)	1 (10.0)	1 (6.2)	0.470
Anticoagulant use, *n* (%)	4 (23.5)	2 (20.0)	3 (18.8)	1.000
Smoking history, *n* (%)	8 (47.1)	6 (60.0)	9 (56.2)	0.793
BCVA, logMAR, mean (SD)	0.31 (0.28)	0.39 (0.39)	0.37 (0.32)	0.818
Anatomical parameters at baseline				
CRT (μm), mean (SD)	257.8 (86.4)	393.1 (182.6)	319.9 (139.7)	0.048
CCT (μm), mean (SD)	251.2 (103.0)	251.1 (103.4)	271.3 (97.5)	0.820
PED height at fovea (μm), mean (SD)	99.0 (185.4)	33.2 (38.0)	54.3 (64.5)	0.375
Disrupted EZ at fovea, *n* (%)	11 (64.7)	8 (80.0)	9 (56.2)	0.514
SRH, within 6 mm, *n* (%)	6 (35.3)	6 (60.0)	6 (37.5)	0.455
SHRM within 6 mm, *n* (%)	10 (58.8)	10 (100)	9 (56.2)	0.030
HRF within 6 mm, *n* (%)	6 (35.3)	7 (70.0)	11 (68.8)	0.098
Intraretinal fluid in fovea, *n* (%)	7 (41.2)	4 (40.0)	3 (18.8)	0.346
Subretinal fluid in fovea, *n* (%)	11 (64.7)	10 (100.0)	15 (93.8)	0.036
Presence of PED in fovea, *n* (%)	12 (70.6)	6 (60.0)	13 (81.2)	0.516
Subtype				0.091
Type 1	8 (47.1)	2 (20.0)	3 (18.8)	
Type 1 with polyps (PCV)	6 (35.3)	7 (70.0)	13 (81.2)	0.022
Type 2	2 (11.8)	1 (10.0)	0 (0.0)	
Type 3 (RAP)	1 (5.9)	0 (0.0)	0 (0.0)	
Clinical course at 1 year				
BCVA (logMAR), mean (SD)	0.17 (0.35)	0.20 (0.48)	0.20 (0.25)	0.974
CRT (μm), mean (SD)	167.6 (37.7)	210.0 (51.1)	191.4 (98.0)	0.295
Intraretinal fluid in fovea, *n* (%)	0 (0.0)	1 (10.0)	1 (6.2)	0.511
Subretinal fluid in fovea, *n* (%)	1 (5.9)	2 (20.0)	3 (18.8)	0.540
Treatment interval ≥12 weeks, *n* (%)	14 (82.4)	9 (90.0)	9 (56.2)	0.140
Treatment interval ≥16 weeks, *n* (%)	13 (76.5)	6 (60.0)	7 (43.8)	0.163
Total number of injections, mean (SD)	5.7 (1.6)	6.3 (0.7)	7.8 (1.1)	0.001
Switched to another drug, *n* (%)	1 (5.9)	0 (0.0)	3 (18.8)	0.339

Values are expressed as the mean (standard deviation) or number (percentage). *p* values are calculated using ANOVA or Fisher’s exact test, as appropriate. CRT: central retinal thickness; CCT: central choroidal thickness; PED: pigment epithelial detachment; EZ: ellipsoid zone; SRH: subretinal hemorrhage; SHRM: subretinal hyperreflective material; HRF: hyperreflective foci; BCVA: best-corrected visual acuity.

**Table 3:** Comparison of baseline characteristics between individuals with successful and failed extension of treatment interval to ≥16 weeks in neovascular age-related macular degeneration.

	Treatment Interval Extended to ≥16 Weeks		
	Success	Failed	*p* values
Number of eyes, *n* (%)	26 (60.5)	17 (39.5)	
Clinicodemographic characteristics			
Age (years), mean (SD)	77.5 (9.4)	75.2 (8.3)	0.403
Sex: Female, *n* (%)	9 (34.6)	3 (17.6)	0.306
Hypertension, *n* (%)	19 (73.1)	10 (58.8)	0.507
Diabetes mellitus, *n* (%)	6 (23.1)	2 (11.8)	0.446
Malignancy, *n* (%)	4 (15.4)	5 (29.4)	0.445
History of cardiovascular events, *n* (%)	5 (19.2)	1 (5.9)	0.376
Anticoagulant use, *n* (%)	7 (26.9)	2 (11.8)	0.281
Smoking history, *n* (%)	13 (50.0)	10 (58.8)	0.756
BCVA (logMAR), mean (SD)	0.32 (0.25)	0.40 (0.40)	0.433
Anatomical parameters at baseline			
CRT (μm), mean (SD)	294.9 (109.3)	339.2 (178.6)	0.317
CCT (μm), mean (SD)	251.3 (105.2)	269.8 (90.9)	0.555
PED height at fovea (μm), mean (SD)	35.3 (41.9)	115.7 (184.8)	0.038
Disrupted EZ at fovea, *n* (%)	13 (50.0)	15 (88.2)	0.020
SRH, within 6 mm, *n* (%)	8 (30.8)	10 (58.8)	0.114
SHRM within 6 mm, *n* (%)	17 (65.4)	12 (70.6)	1.000
HRF within 6 mm, *n* (%)	14 (53.8)	10 (58.8)	1.000
Intraretinal fluid in fovea, *n* (%)	9 (34.6)	5 (29.4)	1.000
Subretinal fluid in fovea, *n* (%)	22 (84.6)	14 (82.4)	1.000
Presence of PED in fovea, *n* (%)	18 (69.2)	13 (76.5)	0.735
Subtype			0.103
Type 1	10 (38.5)	3 (17.6)	
Type 1 with polyps (PCV)	12 (46.2)	14 (82.4)	0.026
Type 2	3 (11.5)	0 (0.0)	
Type 3 (RAP)	1 (3.8)	0 (0.0)	

Values are shown as the mean (standard deviation) or number (percentage). *p* values are calculated using *t*-tests or Fisher’s exact test, as appropriate. CRT: central retinal thickness; CCT: central choroidal thickness; PED: pigment epithelial detachment; EZ: ellipsoid zone; SRH: subretinal hemorrhage; SHRM: subretinal hyperreflective material; HRF: hyperreflective foci; BCVA: best-corrected visual acuity.

## Data Availability

All data were accessible to Machida A., who assumes accountability for ensuring both their integrity and the accuracy of the analysis. Data underlying the findings of this work are available from the corresponding author upon request. Public sharing of the dataset is restricted because of concerns related to participant confidentiality and privacy protection.

## Meeting Presentations

Parts of the data were orally presented at The 63rd Annual Meeting of the Japanese Retina and Vitreous Society, Osaka, Japan, 2024 [35].

## Abbreviations

The following abbreviations are used in this manuscript:

nAMD	Neovascular age-related macular degeneration
VEGF	Vascular endothelial growth factor
AMD	Age-related macular degeneration
MNV	Macular neovascularization
TAE	Treat-and-extend
PED	Pigment epithelial detachment
BCVA	Best-corrected visual acuity
PCV	Polypoidal choroidal vasculopathy
CRT	Central retinal thickness
OCT	Optical coherence
SRF	Subretinal fluid
